# Enhancing Fermented Sausage Quality with *Weissella hellenica*, *Lactobacillus sakei*, and *Pediococcus pentosaceus*

**DOI:** 10.3390/gels12030222

**Published:** 2026-03-09

**Authors:** Yuan Fu, Lingjie Zhang, Hairong Long, Zhitian Yin, Xing Sun, Wen Nie, Qinqing Zhuo, Congyu Lin, Shuangjie Zhu, Yeye Du, Longwei Jiang

**Affiliations:** 1 School of Biological Science and Food Engineering, Chuzhou University, No. 1, West Huifeng Road, Chuzhou 239099, China; 2 College of Food Science and Engineering, Jilin Agricultural University, 2888 Xincheng Street, Changchun 130118, China; 3 Anhui Heat-Sensitive Materials Processing Engineering Technology Research Center, Chuzhou University, Chuzhou 239000, China; 4 Jilin Province Product Quality Supervision and Inspection Institute, Changchun 130103, China; 5 School of Food Science and Technology, Jiangnan University, Wuxi 214122, China; 6 School of Food and Nutrition, Anhui Agricultural University, Hefei 230036, China

**Keywords:** fermented sausage, starter culture, volatile flavor compounds, storage stability

## Abstract

The natural fermentation of sausages often results in inconsistent gel texture and flavor stability. This study introduces a compound fermenter group (*Weissella hellenica*, *Lactobacillus sakei*, and *Pediococcus pentosaceus*) to improve the quality of fermented sausages. The aim was to evaluate its flavor-modulating and quality-preserving effects, addressing the research gap in applying these microbial synergies in fermented meats. Sausages inoculated with the compound fermenter group were compared with control group (naturally fermented) over 90-day frozen storage using gas chromatography–mass spectrometry and physicochemical, microbiological, and sensory analyses. The results showed that the compound fermenter group enhanced protein gel network stability (increased hardness and chewiness; reduced moisture loss), enriched the volatile aroma profile, with an 8.7% increase in the variety of flavor compounds and no detected lipid oxidation-derived off-flavor aldehydes (e.g., trans-2-nonenal), and improved oxidative and microbial stability (lower thiobarbituric acid reactive substances and total volatile basic nitrogen values; total viable counts within safety limits), with consistently higher sensory scores. The compound fermenter group effectively coordinates proteolysis, gelation, and flavor metabolism, offering a promising strategy for producing high-quality fermented meat gels with optimized texture and extended shelf life.

## 1. Introduction

Fermented sausage is a globally popular meat product, valued for its extended shelf life and distinctive flavor profile. Its production involves the fermentation of meat products under controlled conditions, a process traditionally reliant on indigenous microbiota. While natural fermentation persists in small-scale operations, it presents significant challenges for industrial standardization, including inconsistent microbial successions leading to batch-to-batch flavor variability, delayed acidification elevating safety risks, and insufficient endogenous antioxidant activity accelerating lipid oxidation and quality degradation during storage [1,2].

To address these limitations, the development and application of defined starter cultures has become a central research focus. Multi-strain starters, leveraging functional complementarity, have demonstrated superior performance over single strains in enhancing safety, acidification kinetics, proteolysis, and overall sensory quality [3,4]. Consequently, commercial starter cultures predominantly feature lactic acid bacteria (LAB) as the core component. However, this paradigm often overlooks other promising LAB genera with exceptional stress tolerance and functional attributes, such as *Weissella hellenica*, *Lactobacillus sakei*, and *Pediococcus pentosaceus* [5,6]. Notably, systematic investigations into the synergistic potential of these genera—particularly their collective impact on shaping complex flavor profiles and ensuring long-term storage stability—remain scarce, representing a critical knowledge gap. Furthermore, the textural quality and stability of fermented sausages are fundamentally governed by the myofibrillar protein gel network formed during processing [7,8]. The development of this gel is highly sensitive to proteolytic activity, pH decline, and microbial metabolism. Natural fermentation, with its unpredictable microbial succession, often leads to excessive or insufficient proteolysis, resulting in gel weakness, syneresis, and textural defects [7]. Defined starter cultures offer a means to precisely steer these biochemical processes. Selected lactic acid bacteria (LAB) not only ensure safety and acidification but also modulate proteolysis to foster an optimal gel microstructure [9]. However, the specific role and synergistic potential of non-conventional LAB, such as Weissella, in shaping the gel physics and water-holding functionality of fermented sausages remain an underexplored frontier, presenting a significant opportunity for product innovation.

The rational selection of starter strains is paramount. Based on extensive isolation and characterization from fermented meat products, *Weissella hellenica* (Wh), *Lactobacillus sakei* (Ls), and *Pediococcus pentosaceus* (Pp) were identified as candidates with outstanding potential. These strains exhibit robust growth and acidification under stringent processing conditions (e.g., tolerance to pH ~4.2, 6% NaCl, 150 mg/kg NaNO_2_). Critically, they possess complementary functional traits: *W. hellenica* is noted for its prolific production of key flavor volatiles [10] and antioxidant activity [11], and *L. sakei* and *P. pentosaceus* demonstrate broad-spectrum antimicrobial activity against common foodborne pathogens. Furthermore, confirmed compatibility via plate confrontation assays ensures consortium stability. This triad integrates flavor enhancement, oxidative defense, and biopreservation, presenting a holistic strategy to concurrently tackle the dual challenges of flavor inconsistency and storage spoilage.

We therefore hypothesize that a designed ternary consortium of *W. hellenica*, *L. sakei*, and *P. pentosaceus* will significantly improve the flavor complexity and oxidative stability of fermented sausages during extended storage compared to natural fermentation. To test this hypothesis, sausages inoculated with the Wh-Ls-Pp consortium were produced alongside naturally fermented controls. A comprehensive analysis was conducted, employing gas chromatography–mass spectrometry (GC-MS) to characterize volatile flavor compounds and monitoring key physicochemical (pH, TBARS, TVB-N), microbiological, and sensory parameters over a 90-day frozen storage period.

This study aims to: (i) elucidate the flavor-modulating and quality-preserving effects of the novel Wh-Ls-Pp consortium; (ii) bridge the research gap in applying Weissella–Lactobacillus–Pediococcus synergies in fermented meats; and (iii) provide actionable technical insights to advance the development of multifunctional starters for industrial production of high-quality, stable fermented sausage products.

## 2. Results and Discussion

### 2.1. Free Amino Acids

Free amino acids are not only important flavor precursors but also directly influence the taste characteristics of fermented meat products [12]. As shown in Table 1, the total free amino acid content increased from 1091.95 mg/100 g in the control group (CK) to 1187.90 mg/100 g in the compound fermenter group (CF), representing an 8.8% increase. In both groups, leucine was the most abundant free amino acid, followed by cysteine, phenylalanine, and valine, with these four amino acids accounting for approximately 47% of the total free amino acids. The changes in specific free amino acids were closely related to the metabolic activities of individual strains. *Lactobacillus sakei* possesses strong proteolytic activity, being able to hydrolyze myofibrillar and sarcoplasmic proteins to release small peptides and free amino acids [13,14]; in this study, the branched-chain amino acids (leucine, isoleucine, valine) in the CF group were significantly higher than those in the CK group: leucine increased from 201.31 to 228.06 mg/100 g (+13.3%), isoleucine from 83.73 to 91.35 mg/100 g (+9.1%), and valine from 90.66 to 108.36 mg/100 g (+19.5%), which is consistent with the proteolytic activity of *L. sakei* [15]. *P. pentosaceus* also participates in protein degradation and amino acid release [16]; in this study, the sulfur-containing amino acids (cysteine, methionine) in the CF group increased significantly: cysteine from 114.96 to 135.23 mg/100 g (+17.6%) and methionine from 45.53 to 52.56 mg/100 g (+15.4%), which is associated with the enzymatic activity of *P. pentosaceus* [17]. *W. hellenica* mainly contributed to the increase in glutamate and alanine: glutamate increased from 35.93 to 51.37 mg/100 g (+43.0%) and alanine from 73.36 to 90.45 mg/100 g (+23.3%), which is related to its proteolytic and amino acid metabolic capabilities [3,11].

The enriched free amino acid pool has direct sensory implications. Glutamate is a key contributor to umami taste, and its significant increase in the CF group (+43.0%) directly promoted the enhancement of savory flavor [18]. Branched-chain amino acids are important precursors of branched-chain aldehydes (such as 2-methylbutanal and isovaleraldehyde), which were detected only in the CF group sausages (Table 2), imparting fruity and chocolate-like notes to the product [17,19]. Sulfur-containing amino acids can participate in the formation of characteristic meaty flavors through Maillard reactions and Strecker degradation during storage [20]; the significantly higher sulfur-containing amino acid content in the CF group was consistent with its richer volatile flavor characteristics (Table 2). These results indicate that the ternary bacterial consortium enhanced proteolysis and amino acid metabolism through synergistic effects, forming a richer free amino acid pool, thereby improving the taste and aroma quality of fermented sausages.

### 2.2. Volatile Flavor Compounds

Volatile flavor compounds in fermented sausages were analyzed by GC-MS, with the results shown in Table 2. A total of 46 compounds were identified in the CK group, compared to 50 compounds in the CF group, indicating that the compound fermenter increased the variety of flavor substances by 8.7% [20]. The two groups shared 24 common compounds, mainly including terpenes, aldehydes, and ethers. Among these, β-pinene (CK: 9.72%, CF: 4.48%) and α-pinene (CK: 8.35%, CF: 2.80%) were the predominant terpenes. The CF group exhibited significantly higher contents of benzaldehyde (4.35% vs. 1.55%), eleganin (8.73% vs. 3.23%), and nutmeg ether (3.48% vs. 2.21%) compared to the CK group, while N-hexanal content was lower in the CF group (5.28% vs. 7.51%).

The CK group had 22 unique compounds, including trans-2-nonenal (0.52%) and trans-2-decenal (1.01%), which are characteristic products of lipid oxidation associated with off-flavors such as rancidity [21]. The CF group contained 26 unique compounds, most of which were active flavor substances with pleasant aromas. Branched-chain aldehydes (2-methylbutanal 0.77%, isovaleraldehyde 1.13%) were detected exclusively in the CF group; these are characteristic products of microbial metabolism of branched-chain amino acids, imparting fruity and chocolate-like notes [20]. Terpene alcohols, including (-)-4-terpenol (8.92%) and α-terpineol (1.66%), exhibited pine and floral characteristics [22,23].

Notably, the compound fermenter significantly altered the compositional profile of flavor substances: the relative contents of key off-flavor compounds associated with lipid oxidation—namely hexanal, β-pinene, and benzaldehyde—decreased by over 97% in the CF group, demonstrating that the compound fermenter effectively inhibited lipid oxidation processes. These flavor changes were closely associated with specific microbial activities: *W. hellenica* dominated the metabolism of branched-chain amino acids to generate branched-chain aldehydes [12]; *L. sakei* and *P. pentosaceus* participated in the conversion of terpenes to generate terpene alcohols [24]; and the compound fermenter completely eliminated off-flavor aldehydes through competitive inhibition [25]. Quantitative analysis revealed that the CF group exhibited an 8.7% increase in total flavor compounds, an 18.2% increase in unique compounds, complete suppression of off-flavor aldehydes, and de novo generation of beneficial branched-chain aldehydes and terpene alcohols. These results demonstrate that the ternary bacterial consortium achieved targeted “flavor modulation” through the directed regulation of metabolic pathways, significantly enhancing the flavor quality of fermented sausages.

### 2.3. pH and Moisture Content

**Figure 1:**
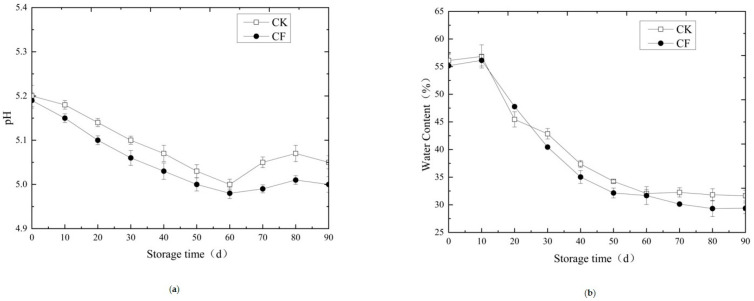
Changes in pH and water content of fermented sausage during storage. (**a**) changes in the pH of fermented sausage during storage; (**b**) changes in the water content of fermented sausage during storage; CK: naturally matured without adding mixed strains (control group, CK); CF: sausages with mixed strains (compound fermenter group, CF).

The pH dynamics during storage followed a characteristic pattern: an initial decrease followed by a gradual increase (Figure 1a). The initial drop (Days 0–30) is attributed to the continued production of lactic and acetic acids by lactic acid bacteria. The subsequent rise results from the accumulation of alkaline compounds, such as ammonia and amines, from protein degradation, which buffer the system [24]. While the initial pH was similar between groups (CK: 5.20, CF: 5.19), the CF group maintained a more stable pH throughout storage (5.1–5.2), remaining within the acceptable range for fermented sausages. This enhanced pH stability is attributed to the balanced metabolic activities of the ternary consortium, which regulates acid production and prevents excessive pH fluctuation [19].

Water content progressively decreased in both groups due to water evaporation and redistribution within the matrix (Figure 1b). After 90 days, the CF group exhibited a lower final moisture content (29.38%) compared to CK (31.65%). This difference is directly linked to the structural properties of the protein gel network. The CF group developed a more extensive and cohesive myofibrillar protein gel during fermentation, as evidenced by increased hardness and chewiness (Table 3). While this denser network initially enhances water-holding capacity by entrapping water more effectively, it undergoes different shrinkage dynamics during long-term frozen storage [7,8]. Ice crystal formation and recrystallization within the more organized gel matrix may lead to slightly higher moisture loss, representing a trade-off between improved texture and moisture retention that is characteristic of well-formed protein gels in fermented meats [9].

### 2.4. Color

The color parameters of fermented sausages during storage are summarized in Table 3. Both groups showed an initial increase in lightness (L) and redness (a), corresponding to nitrosylmyoglobin formation under low-pH conditions. Subsequently, L* and a* values gradually declined due to myoglobin oxidation, moisture loss, and fat oxidation [3].

The rate of color degradation, particularly the decline in a* values, was notably slower in the CF group compared to CK. This enhanced color stability is directly linked to the antioxidant properties of *W. hellenica* within the ternary consortium [7]. W. hellenica produces antioxidant enzymes, including catalase and superoxide dismutase, which scavenge free radicals and inhibit lipid oxidation [17]. By reducing oxidative stress in the meat matrix, these activities protect myoglobin from oxidation, slowing the conversion of red oxymyoglobin to brown metmyoglobin.

The practical significance of these color changes is substantial. Redness (a) is a key determinant of consumer acceptance for fermented sausages, directly influencing purchase decisions. The slower decline in the CF group extends the product’s visual shelf-life and maintains retail appeal. Furthermore, color stability serves as an indirect indicator of oxidative status; the better color retention in CF group aligns with its lower TBARS values (Section 2.5), confirming reduced lipid oxidation. These improvements enhance commercial value by minimizing product rejection due to discoloration during storage

### 2.5. Texture Profile Analysis

The texture parameters of fermented sausages changed significantly during storage (Table 4). Both groups exhibited an initial increase in hardness and chewiness, peaking around day 50–60, followed by a gradual decline. The initial increase is attributed to protein denaturation and gel network strengthening under conditions of lower pH and reduced moisture content. The subsequent softening results from prolonged proteolytic activity, which gradually degrades the protein matrix [7].

Notably, the textural changes were less pronounced in the CF group, which maintained higher hardness and chewiness values throughout late storage. At day 90, the CF group exhibited a hardness of 298.24 N compared to 242.1 N in CK, and chewiness of 523.09 mJ compared to 421.345 mJ in CK. This improved texture retention can be mechanistically linked to the consortium’s activities: the ternary consortium promotes controlled acidification and proteolysis during fermentation, leading to a more stable and cohesive myofibrillar protein gel network [8]. This denser structure provides greater resistance to deformation. The consortium balances proteolytic enzyme activity, preventing excessive protein degradation that causes texture softening [9]. This is evidenced by the CF group’s sustained hardness values even at advanced storage stages. The more organized gel matrix in CF group entraps water more effectively, contributing to sustained texture properties throughout storage [15].

The practical significance of these texture improvements is substantial. Hardness directly influences slice ability and mouthfeel, key quality attributes for consumer acceptance of fermented sausages. Chewiness correlates with the energy required for mastication, affecting eating quality and satiety perception. The CF group’s ability to maintain superior texture at day 90 (hardness: +23.2%, chewiness: +24.1% vs. CK) translates to extended textural shelf-life and maintained product quality during distribution. These improvements enhance commercial value by reducing texture-related quality defects and extending product acceptability.

### 2.6. Lipid Oxidation and Total Volatile Basic Nitrogen

**Figure 2:**
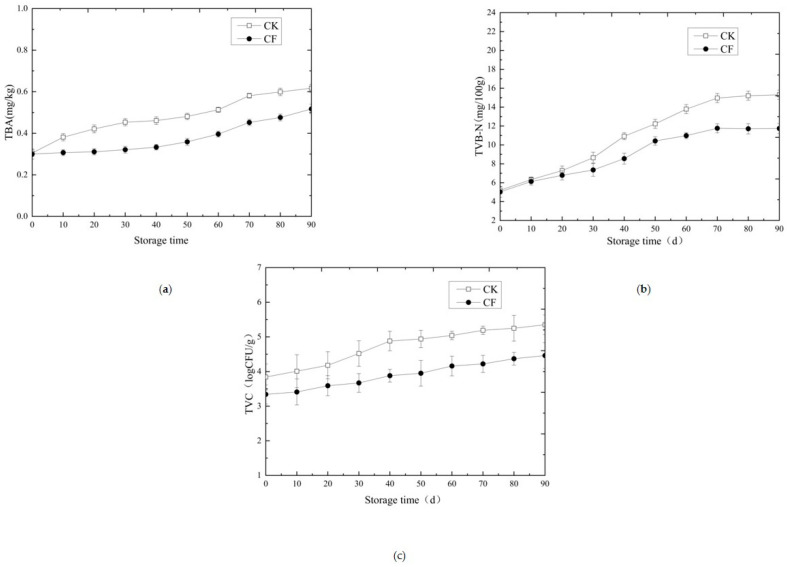
Changes in TBA, TVB-N and TVC of fermented sausage during storage. (**a**) Changes in the TBA of fermented sausage during storage; (**b**) changes in TVB-N of fermented sausage during storage; (**c**) changes in TVC of fermented sausage during storage; CK: naturally matured without adding mixed strains (control group, CK); CF: sausages with mixed strains (compound fermenter group, CF).

The thiobarbituric acid reactive substances (TBARS) value, an indicator of lipid oxidation, remained below the critical limit of 1.0 mg MDA/kg throughout storage for both groups [25] (Figure 2a). However, the CF group consistently exhibited significantly lower TBARS values, reaching 0.517 mg/kg at day 90 compared to 0.617 mg/kg in CK (a 16.2% reduction). This superior antioxidant capacity is mechanistically linked to the consortium’s activities. *W. hellenica* produces antioxidant enzymes (catalase, superoxide dismutase) that scavenge free radicals and inhibit lipid peroxidation [17]. *P. pentosaceus* contributes through its metal-chelating capacity and reducing power [16]. The reduced lipid oxidation directly correlates with the absence of off-flavor aldehydes (trans-2-nonenal, trans-2-decenal) in the CF group (Table 2).

Similarly, total volatile basic nitrogen (TVB-N), an indicator of protein breakdown and spoilage, increased over time but remained well within the safety limit of 20 mg/100 g [26] (Figure 2b). The CF group demonstrated a significantly slower increase, culminating in a final value of 11.74 mg/100 g—23.3% lower than the CK group (15.31 mg/100 g). This suppression of spoilage metabolites results from multiple mechanisms: (i) rapid acidification by *L. sakei* and *P. pentosaceus* suppresses proteolytic spoilage bacteria [13]; (ii) competitive exclusion limits substrate availability; (iii) potential bacteriocin production by *P. pentosaceus* directly inhibits spoilage organisms [16].

### 2.7. Microbiological Safety

The total viable count (TVC) increased during storage in both groups (Figure 2c). However, the growth kinetics differed dramatically. While the TVC in the CK group exceeded the safety limit of 5 log CFU/g [27,28] by day 90, the TVC in the CF group remained below this threshold throughout the 90-day study. This unequivocally demonstrates the biopreservative efficacy of the consortium. Mechanistically, the consortium’s rapid establishment and dominance in the microbial ecosystem, combined with acid production creating a selective environment, effectively suppressed background and spoilage microbiota. Additionally, *P. pentosaceus* may produce antimicrobial compounds that directly inhibit competitors [20].

### 2.8. Sensory Evaluation

**Figure 3:**
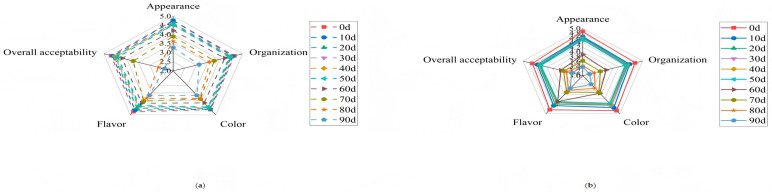
Changes in control group and compound fermenter group sensory score of fermented sausage during storage. (**a**) Naturally matured without adding mixed strains (control group, CK); (**b**) sausages with mixed strains (compound fermenter group, CF).

Sensory scores for all attributes gradually declined during storage (Figure 3), a common trend due to oxidative and microbial changes [29]. However, the CF group received consistently and significantly higher scores (*p* < 0.05) than the CK group for flavor, texture, color, and overall acceptability at every time point. Panelists noted a more intense, balanced, and pleasant fermented flavor and a firmer, more appealing texture in CF sausages.

The superior sensory performance of the CF group is a direct integrative consequence of the improvements described above. The 16.2% reduction in TBARS correlates with the absence of lipid oxidation-derived off-flavors, directly enhancing flavor scores. The 23.3% lower TVB-N indicates reduced protein degradation, preserving fresh meaty flavor and preventing ammonia-like off-notes. The maintained TVC below safety limits ensures microbiological safety, indirectly supporting sensory quality by preventing spoilage-related texture and flavor deterioration. Together, these biochemical and microbiological improvements translate into the consistently superior sensory attributes of the CF group throughout storage.

## 3. Conclusions

This study demonstrates that the ternary consortium consisting of *Weissella hellenica*, *Lactobacillus sakei*, and *Pediococcus pentosaceus* functions as an effective multifunctional starter culture, simultaneously improving the gel texture, flavor characteristics, and shelf life of fermented sausages. Compared with previous studies on lactic acid bacteria consortia [12,15,16], this work is the first to systematically reveal the multiple functionalities of Wh-Ls-Pp in fermented meat products. Beyond enriching the volatile flavor profile and inhibiting spoilage, the most significant contribution of this consortium lies in its capacity to construct a superior protein gel network. This was evidenced by the enhanced textural properties (increased hardness and chewiness), improved water retention, and better color stability throughout frozen storage. The metabolic synergy among the strains enables the fine-tuned regulation of proteolysis and the formation of a more cohesive gel matrix, directly addressing the textural inconsistencies inherent to natural fermentation.

The findings of this study are consistent with those of Wang et al. [12], who reported that LAB inoculation improves the quality of dry sausages, and further extend the understanding of flavor regulation by mixed starter cultures, as reported by Chen et al. [20] and Li et al. [21]. Compared with the studies by Casaburi et al. [15] and Park et al. [17], this work not only confirms the proteolytic and antioxidant functions of individual strains but also reveals their synergistic effects when combined as a consortium. Nevertheless, certain limitations should be acknowledged. First, although the 90-day frozen storage period covers typical commercial shelf life, the quality evolution over extended storage (e.g., 6–12 months) remains to be investigated. Second, the synergistic mechanisms among the three strains in the consortium were primarily inferred based on functional characteristics; future studies employing multi-omics approaches (e.g., metagenomics, transcriptomics) are needed to elucidate the underlying molecular regulatory networks. Additionally, the performance of the consortium may vary with different raw material batches and processing conditions, warranting further validation of its stability and applicability at pilot and industrial scales.

In conclusion, the Wh-Ls-Pp consortium provides a robust microbial toolkit for the rational design of fermented meat gels, enabling predictable high-quality sensory attributes and extended stability, with clear translational potential for the meat processing industry. This study not only fills the research gap regarding Wh-Ls-Pp synergies in fermented meat products but also offers new insights into the functional design of multi-strain starter cultures.

## 4. Materials and Methods

### 4.1. Materials and Chemicals

Fresh pork (lean-to-fat ratio 9:1, *w*/*w*) was obtained from a local supplier. *W. hellenica* (isolated from Kunming beef jerky), *L. sakei* (isolated from Nanjing Duck), and *P. pentosaceus* (isolated from Sichuan pickle) were previously isolated, identified, and preserved by the Laboratory of Livestock Product Processing, Jilin Agricultural University. De Man, Rogosa and Sharpe (MRS) broth was used for strain cultivation. Edible collagen casings (diameter 50 mm) were used for stuffing. Food-grade ingredients, including sugar, sodium chloride, sodium nitrite, sodium tripolyphosphate, monosodium glutamate, carrageenan, white pepper powder, cardamom powder, and L-ascorbic acid, were used for sausage formulation. All chemicals and reagents were of analytical grade and purchased from Changchun Yibo Biotechnology Co., Ltd. (Changchun, China).

### 4.2. Sausage Preparation and Processing

#### 4.2.1. Starter Culture Preparation

Each strain (*W. hellenica*, *L. sakei*, and *P. pentosaceus*) was individually activated in MRS broth at 37 °C for 18–24 h. Cells were harvested by centrifugation (4000× *g*, 10 min, 4 °C), washed twice with sterile saline (0.85% NaCl), and resuspended in sterile saline to achieve a final concentration of approximately 10^9^ CFU/mL. A composite starter culture (CF) was prepared by mixing the cell suspensions of *W. hellenica*, *L. sakei*, and *P. pentosaceus* at a volumetric ratio of 2:1:1.

#### 4.2.2. Formulation and Processing

The basic sausage batter was prepared by homogenizing minced fresh pork with the following ingredients (*w*/*w* based on meat weight): sugar 1.3%, NaCl 2.0%, sodium nitrite 0.01%, sodium tripolyphosphate 0.3%, monosodium glutamate 0.1%, carrageenan 0.3%, white pepper powder 0.5%, cardamom powder 0.1%, and L-ascorbic acid 0.08%. An appropriate amount of ice water was added to facilitate mixing. The batter was divided into two portions. For the control group (CK, natural fermentation), no starter culture was added. For the treatment group (CF), the composite starter culture suspension was inoculated at a level of 10^7^ CFU/g batter. Each batter portion was stuffed into collagen casings to form sausages (approximately 10 cm in length and 5 cm in diameter). The sausages were rinsed with warm water and then subjected to fermentation in a climate chamber at 37 °C and 85–90% relative humidity for 15–21 h, until the pH dropped to 4.8–5.0. Subsequently, the sausages were dried in an electric oven at 50 °C for 6 h, followed by maturation in a climate chamber at 14–15 °C and 75–80% relative humidity for 72 h [30,31].

#### 4.2.3. Storage

After processing, all sausages were cooled to room temperature (25 ± 1 °C), vacuum-packaged in food-grade bags, and stored at −20 ± 1 °C in a constant-temperature freezer (Model BCD-580WDPU, Midea, Hefei, China) for up to 90 days. This temperature aligns with FAO recommendations for long-term meat storage [32]. Samples were withdrawn at 10-day intervals for analysis.

### 4.3. Free Amino Acids Analysis

Free amino acid content was determined according to a published method with slight modifications [33]. Briefly, 0.2 g of sample was hydrolyzed with 10 mL of 6 mol/L HCl at 110 °C for 24 h. The hydrolysate was cooled, diluted to 50 mL, and filtered. A 10 mL aliquot was evaporated to dryness, and the residue was reconstituted in 0.1 mol/L HCl. After derivatization with an internal standard, the solution was filtered (0.22 μm aqueous membrane) and analyzed using high-performance liquid chromatography (Shimadzu 20AT, Kyoto, Japan) equipped with a PDA detector and a C_18_ column (4.6 mm × 150 mm, 3 μm). The detection wavelength was 338 nm, the column temperature was 50 °C, and the flow rate was 1.6 mL/min.

### 4.4. Volatile Compound Analysis

Volatile compounds were analyzed by headspace solid-phase microextraction coupled with gas chromatography–mass spectrometry (HS-SPME/GC–MS), following an established method with modifications [34]. Homogenized sample (2 g) was placed in a 20 mL vial with 15% NaCl (*w*/*v*). After equilibration at 50 °C for 10 min, volatiles were extracted using a 50/30 μm DVB/CAR/PDMS fiber at 45 °C for 40 min. Analysis was performed on an Agilent 7890B GC system (Agilent Technologies, Santa Clara, CA, USA) coupled with a 7000D mass spectrometer (Agilent Technologies, Santa Clara, CA, USA). Separation was achieved on an HP-5MS column (30 m × 0.25 mm × 0.25 μm). The oven temperature program was: 40 °C for 3 min, which was increased to 85 °C at 8 °C/min, then to 150 °C at 10 °C/min and held for 2 min, before finally being ramped to 250 °C at 15 °C/min and held for 5 min. Helium was used as the carrier gas at 1.0 mL/min. Mass spectra were acquired in the scan range of *m*/*z* 35–500. Compounds were tentatively identified by matching against the NIST05 library (similarity ≥ 80%) and relative contents were calculated by peak area normalization.

### 4.5. Physicochemical Analyses

#### 4.5.1. pH

Measured according to GB 5009.237-2016 [35]. A 5 g sample was homogenized with 50 mL of distilled water, and the pH of the filtrate was determined.

Moisture content: Determined using a calibrated moisture analyzer (Rotronic, Bassersdorf, Switzerland).

#### 4.5.2. Color

Measured using a HunterLab ColorFlex system (8 mm aperture, D65 illuminant, 10° observer). Slices were placed in a sample cup, and L* (lightness), a* (redness), and b* (yellowness) values were recorded at four rotational angles.

#### 4.5.3. Texture Profile Analysis (TPA)

Hardness and chewiness were determined using a texture analyzer (TMA-Pro, FTC, Springfield, VA, USA) in TPA mode. Cylindrical samples (1.5 cm diameter × 1 cm height) were compressed to 50% of their height at a speed of 60 mm/min with a 2 s interval between cycles [36].

#### 4.5.4. Thiobarbituric Acid Reactive Substances (TBARS)

Lipid oxidation was assessed as described previously with modifications [37]. Results were expressed as mg malondialdehyde (MDA) per kg sample.

#### 4.5.5. Total Volatile Basic Nitrogen (TVB-N)

Determined by the semi-micro Kjeldahl method according to GB 5009.228-2016 [38]. Results were expressed as mg N per 100 g sample.

### 4.6. Microbiological Analysis

Total viable count (TVC) was enumerated following the Chinese National Food Safety Standard GB 4789.2-2022 [39]. A 10 g sample was homogenized with 90 mL of sterile saline (0.85% NaCl), serially diluted, and plated on Plate Count Agar. Plates were incubated at 37 °C for 48 h, and results were expressed as log_10_ CFU/g.

### 4.7. Sensory Evaluation

Sensory analysis was performed by a trained panel of thirty members from the College of Food Science and Engineering, Jilin Agricultural University. Panelists were selected and trained according to established sensory science protocols [33]. Oral informed consent was obtained prior to participation. Samples were heated uniformly, sliced, and presented in a randomized order. Appearance, texture, color, flavor, and overall acceptability were evaluated using a 5-point hedonic scale (1 = dislike extremely; 5 = like extremely).

### 4.8. Statistical Analysis

All experiments followed a completely randomized design and were performed in triplicate. Data were expressed as mean ± standard deviation. Statistical analyses were conducted using SPSS software (Version 22.0, IBM Corp., Armonk, NY, USA). Prior to analysis, the normality of data distribution was verified using the Shapiro–Wilk test, and homogeneity of variances was confirmed using Levene’s test. All datasets met the assumptions of normality (*p* > 0.05) and homogeneity of variance (*p* > 0.05). Potential outliers were assessed using Grubbs’ test, and no significant outliers were detected (*p* > 0.05); therefore, all data points were retained. One-way analysis of variance (ANOVA) followed by Duncan’s multiple range test was employed to determine significant differences between groups. Statistical significance was set at *p* < 0.05.

## Figures and Tables

**Table 1:** Comparison of free amino acids in control and compound fermenter groups.

Amino Acid	Group	
	CK (mg/100 g)	CF (mg/100 g)
Aspartate	5.80 ± 0.06 ^e^	5.91 ± 0.57
Serine	9.35 ± 5.08 ^e^	18.85 ± 0.67 ^i^
Glutamate	35.93 ± 5.94 ^cde^	51.37 ± 9.05 ^efghi^
Glycine	17.72 ± 2.77 ^de^	21.49 ± 1.17 ^hj^
Histidine	16.46 ± 0.89 ^de^	19.49 ± 0.57 ^i^
Taurine	78.16 ± 15.88 ^cde^	80.07 ± 9.43 ^cdefgh^
Arginine	31.18 ± 0.24 ^cde^	37.56 ± 11.68 ^fghi^
Threonine	25.44 ± 4.76 ^cde^	29.38 ± 2.12 ^ghi^
Alanine	73.36 ± 21.66 ^cde^	90.45 ± 7.05 ^dfg^
Proline	62.43 ± 13.12 ^cde^	69.60 ± 3.60 ^defghi^
Cysteine	114.96 ± 8.66 ^bc^	135.23 ± 26.46 ^c^
Tyrosine	66.9 ± 11.01 ^cde^	61.03 ± 14.60 ^defghi^
Valine	90.66 ± 24.03 ^cde^	108.36 ± 0.21 ^cde^
Methionine	45.53 ± 8.78 ^cde^	52.56 ± 0.28 ^efghi^
Lysine	25.16 ± 1.19 ^cde^	24.76 ± 4.81 ^hi^
Isoleucine	83.73 ± 23.41 ^cde^	91.35 ± 1.47 ^cdef^
Leucine	201.31 ± 62.43 ^b^	228.06 ± 0.56 ^b^
Phenylalanine	107.81 ± 24.71 ^cd^	112.34 ± 0.48 ^cd^
Total free amino acids	1091.93 ± 48.83 ^a^	1187.90 ± 54.53 ^a^

Note: Mean ± standard deviation, parallel three times for each sample. Different lowercase letters indicate significant differences in data (*p* < 0.05). CK: naturally matured without adding mixed strains (control group, CK); CF: sausages including mixed strains (compound fermenter group, CF) (Wh:Ls:Pp = 2:1:1).

**Table 2:** Analysis of flavor compounds in fermented sausages from control and compound fermenter groups.

Shared Flavor Substances			
Serial Number	Flavor Substances	CK Area%	CF Area%
1	N-hexanal	7.51	5.28
2	Hexamethylcyclotrisiloxane	1.75	0.85
3	Heptanal	0.84	0.74
4	α-Pinene	8.35	2.80
5	Benzaldehyde	1.55	4.35
6	β-Pinene	9.72	4.48
7	Mushroom alcohol	1.24	1.46
8	2,5-Octadione	0.52	0.69
9	Laurene	4.86	3.66
10	Octamethylcyclotetrasiloxane	1.29	1.24
11	α-water celery ene	1.02	1.86
12	O-isopropylbenzene	3.75	2.39
13	Nonanal	1.01	2.15
14	Phenylacetaldehyde	1.04	0.55
15	Acetic acid	0.33	0.45
16	Safrole	1.70	2.94
17	Tetradecane	0.63	1.20
18	Methyl eugenol	0.66	1.57
19	N-pentadecane	0.34	0.45
20	Nutmeg ether	2.21	3.48
21	Eleganin	3.23	8.73
22	4-Carene	1.82	1.54
23	2-Methylcyclohexanol	3.73	4.93
24	Turpentene/γ-Turpentene	1.94	3.14
**Unique CK Flavor Substances**			
**Serial Number**	**Flavor Substances**	**CK Area%**	**CF Area%**
1	Glutaraldehyde	0.43	-
2	α-Platycladyne	5.17	-
3	Cis-1-ethyl-2-methylcyclopentane	0.39	-
4	3-Methylnonane	0.39	-
5	3-isopropyl-6-methyl-1-cyclohexene	6.99	-
6	n-octanal	0.85	-
7	Dipentene	8.12	-
8	2,5-Dimethyldodecane	0.40	-
9	6-methylene bicyclic [3.2.0] heptan-3-ene	0.39	-
10	2,6,7-trimethyldecane	4.54	-
11	2,7,10-trimethyldodecane	0.37	-
12	Cis-2-methylcyclohexanol	3.73	-
13	Trans-2-Nonenal	0.52	-
14	Octyl cyclopropane	0.35	-
15	5-ethyldecane	0.84	-
16	Terpinen-4-ol	4.68	-
17	Dodecane	0.64	-
18	Trans-2-decenenal	1.01	-
19	(6E)-2,6-Dimethyloct-2,6-diene	0.50	-
20	3,8-Dimethyldecane	0.84	-
21	(-)-Alpha piperidine	0.66	-
22	Cis-2,4,5-trimethoxy-1-propenylbenzene	0.51	-
**Unique CF Flavor Substances**			
**Serial Number**	**Flavor Substances**	**CK Area%**	**CF Area%**
1	Isovaleraldehyde	-	1.13
2	2-Methylbutanal	-	0.77
3	Toluene	-	0.40
4	2,4-dimethylbenzo [h] quinoline	-	2.01
5	Bicyclic [3.1.0] hex-2-ene	-	2.68
6	Junipene	-	3.99
7	β-Hemihydroquinone	-	4.75
8	2,3,8-trimethyldecane	-	0.56
9	Cis equation-β-Pine oil alcohol	-	0.47
10	4,7-Dimethylundecane	-	0.71
11	2-Carene	-	1.63
12	1-(4-Nitrophenyl)-3-phenylamino propylene ketone	-	0.41
13	1,1-diethylcyclopropane	-	0.39
14	Decane ether	-	0.68
15	Carbonic acid, decyl ester	-	0.70
16	3-Methyldodecane	-	1.04
17	(-)-4-terpenoid alcohol	-	8.92
18	α-Terpineol	-	1.66
19	N-dodecane	-	1.33
20	Decanal	-	0.50
21	(Z)-Decane-2-enal	-	0.83
22	α-Ethylene-phenylacetaldehyde	-	0.69
23	2,6,10-trimethyldodecane	-	0.38
24	Octadecane	-	0.99
25	Heptanyl trifluoroacetic acid	-	0.42
26	β-Caryophyllene	-	0.42

Note: CK: naturally matured without adding mixed strains (control group, CK); CF: sausages including mixed strains (compound fermenter group, CF) (Wh:Ls:Pp = 2:1:1).

**Table 3:** Changes in color of fermented sausages in control and compound fermenter groups.

Day	CK			CF		
	L *	a *	b *	L *	a *	b *
0	60.18 ± 0.23 ^f^	6.22 ± 0.22 ^d^	10.94 ± 0.19 ^a^	60.59 ± 0.21 ^a^	7.44 ± 0.22 ^f^	9.60 ± 0.21 ^f^
10	60.57 ± 0.45 ^a^	6.50 ± 0.14 ^d^	10.62 ± 0.13 ^ef^	60.96 ± 0.23 ^d^	7.08 ± 0.18 ^ef^	9.86 ± 0.34 ^ef^
20	60.96 ± 0.37 ^de^	7.08 ± 0.15 ^bc^	11.86 ± 0.11 ^b^	60.15 ± 0.43 ^f^	7.77 ± 0.28 ^bc^	9.90 ± 0.032 ^ef^
30	61.31 ± 0.28 ^e^	7.66 ± 0.20 ^cd^	11.46 ± 0.12 ^c^	61.95 ± 0.23 ^a^	8.02 ± 0.27 ^de^	10.02 ± 0.13 ^de^
40	61.44 ± 0.35 ^cde^	7.48 ± 0.27 ^abc^	11.33 ± 0.10 ^c^	61.26 ± 0.38 ^ef^	8.10 ± 0.19 ^cd^	10.13 ± 0.18 ^e^
50	61.88 ± 0.39 ^bcd^	7.39 ± 0.32 ^bc^	11.14 ± 0.12 ^cd^	61.96 ± 0.29 ^e^	8.55 ± 0.08 ^ab^	10.74 ± 0.052 ^ab^
60	62.38 ± 0.42 ^bc^	7.77 ± 0.30 ^abc^	11.31 ± 0.086 ^de^	61.27 ± 0.36 ^cd^	8.36 ± 0.15 ^bc^	11.21 ± 0.10 ^bc^
70	62.68 ± 0.46 ^b^	7.82 ± 0.15 ^abc^	10.84 ± 0.10 ^e^	62.33 ± 0.39 ^a^	8.67 ± 0.12 ^a^	11.34 ± 0.023 ^bc^
80	62.24 ± 0.31 ^e^	7.90 ± 0.25 ^a^	11.15 ± 0.079 ^f^	62.14 ± 0.43 ^b^	8.31 ± 0.13 ^cd^	11.21 ± 0.14 ^de^
90	62.39 ± 0.38 ^bc^	7.45 ± 0.16 ^ab^	10.97 ± 0.14 ^cde^	62.45 ± 0.31 ^bc^	8.59 ± 0.14 ^a^	11.14 ± 0.13 ^bc^

Note: Mean ± standard deviation, parallel three times for each sample. Different lowercase letters indicate significant differences in data (*p* < 0.05). CK: naturally matured without adding mixed strains (control group, CK); CF: sausages with mixed strains (compound fermenter group, CF) (Wh:Ls:Pp = 2:1:1); L*: lightness, a*: redness, b*: yellowness.

**Table 4:** Changes in the texture of fermented sausages in control and experimental groups.

Day	CK		CF	
	Hardness (N)	Chewiness (mJ)	Hardnes (N)	Chewiness (mJ)
0	231.5 ± 4.15 ^g^	330.025 ± 5.38 ^g^	228.00 ± 5.16 ^e^	336.07 ± 5.13 ^f^
10	247.1 ± 3.93 ^e^	377.895 ± 6.64 ^e^	247.10 ± 4.81 ^d^	348.75 ± 8.0 ^6f^
20	262.1 ± 4.11 ^ef^	413.025 ± 6.89 ^d^	250.50 ± 5.52 ^d^	337.88 ± 3.55 ^f^
30	274.1 ± 4.42 ^d^	433.115 ± 7.79 ^c^	286.40 ± 4.92 ^c^	392.82 ± 9.70 ^e^
40	284.23 ± 4.03 ^c^	467.245 ± 8.0 ^2c^	284.23 ± 5.50 ^c^	435.13 ± 5.63 ^d^
50	314.61 ± 4.06 ^b^	579.385 ± 9.51 ^a^	303.56 ± 4.47 ^c^	498.06 ± 1.67 ^b^
60	365.58 ± 3.84 ^a^	478.425 ± 2.65 ^b^	312.62 ± 3.01 ^ab^	463.55 ± 5.97 ^c^
70	255.43 ± 3.95 ^fg^	501.365 ± 4.05 ^b^	298.31 ± 7.81 ^bc^	510.17 ± 6.92 ^b^
80	305.32 ± 4.27 ^c^	465.715 ± 5.11 ^c^	304.41 ± 5.66 ^c^	483.12 ± 7.98 ^c^
90	242.1 ± 3.86 ^efg^	421.345 ± 6.45 ^c^	298.24 ± 2.92 ^c^	523.09 ± 5.04 ^b^

Note: Mean ± standard deviation, parallel three times for each sample. Different lowercase letters indicate significant differences in data (*p* < 0.05). CK: naturally matured without adding mixed strains (control group, CK); CF: sausages with mixed strains (compound fermenter group, CF) (Wh:Ls:Pp = 2:1:1).

## Data Availability

Data are contained within the current manuscript.
